# Children's Teeth as Indicators of Disturbed Nutrition

**Published:** 1897-12

**Authors:** S. Henry Dessau


ARTICLE VII.

### CHILDREN'S TEETH AS INDICATORS OF DISTURBED NUTRITION.

BY S. HENRY DESSAU, M. D.

There are many forms of disturbed nutrition in young children where we find the teeth influenced the same as other parts of the body. Such, for example, are rickets, syphilis, diabetes, and the gouty diathesis, manifested in children as chronic intestinal indigestion. There is also a form of cretinism or idiocy, commonly called backward development, which is often of rachitic origin. Of all these mentioned, rickets may be taken as the most familiar illustration of disturbed nutrition affecting the teeth.

Rickets is commonly regarded as a diseased manifestation of faulty nutrition. By this much more is implied than a mere insufficiency of proper quality of food. Nutrition in its broader sense means food suitable to the physiological requirements of the age and condition of the individual. For example, Trousseau, the great French physician, claimed that he had produced rickets in young puppies and pigs by feeding them on raw beef, a food wholly unsuited to their age, though as we all know, dogs are carnivorous and hogs omnivorous animals. The mother's milk of a standard quality is the natural food of infants, but if they are fed on starchy food or a poor quality of milk, or given too much sugar or meat, they may easily become subjects of rickets. But nutrition has an equally important meaning in the proper assimilation of food. Fothergill said that above every nursery door should be written. "It is not what is swallowed, but is assimilated, that nourishes." By assimilation we mean the final and complete conversion of the food into its various elements by the animal organism, and the appropriation of these elements by the various tissue-cells to form new growth. The medium of its performance is through the blood, which receives in exchange those elements called waste matter that have already performed their part in the nourishment of the cell. It will at once be inferred that assimilation depends largely on something more than gastric and intestinal digestion.

Rickets in infants fed on artificial foods, containing chiefly starches, or milk with a small percentage of fat and a large percentage of casein. Such foods do in time undoubtedly derange infantile gastric digestion. But, on the other hand, we see cases of rickets in infants who are breast-fed, the mother's milk being of good average quality, with every reason to believe that it is well digested, so far as the stomach is concerned. Then again, there are rare cases of pre-natal rickets. Hence we infer that assimilation, for its perfect performance, involves complete oxidation in the tissues of the various elements of nutrition in the food, and this requires a free supply of oxygen to the blood in the form of pure air. It has been well said that rickets appears to be one of the penalties of civilization. It is certainly more commonly seen in large cities where breathing space is limited, and is rare or unknown among savage tribes, or those people who live an outdoor life. In this connection, it is interesting to note that rickets is extremely rare in the full-blooded African race as seen in this country, while in the mulatto or hybrid African it is quite common.

Besides proper food and its complete assimilation, nutrition requires as a factor the influence of sunlight, which in some unknown way, probably through furnishing heat, bestows vital energy to the tissues. The growing infant requires an amount of heat which it is unable to generate through its own muscular exercise, and therefore clothing properly adapted to the climate and season are necessary for its due maintenance. As will be shown later on, fats, which are known as respiratory or calorifacient food, aid in maintaining the body heat, and are highly beneficial in correcting the condition producing rickets.

Although rickets is called a disease of malnutrition, it does not necessarily cause wasting of the soft tisues of the body, though in fact these do become flabby, while at the same time the color of the skin becomes pale from a deficiency of the hemoglobin of the blood. This may possibly be explained by the gradual manner of development of the trouble, for in cases of acute malnutrition in infants, where the digestive functions are principally deranged, there is diarrhœa, followed by extreme wasting, commonly called marasmus, which condition does not present any of the phenomena of rickets. One other point in relation to the disturbance of nutrition as being the foundation of rickets is the effect of the mother's condition of health during pregnancy on the future child. The opinion has been advanced that a condition of anemia or poor quality of blood in the mother will so affect the developing embryo that after birth it will be readily disposed to manifest rickets from the slightest causes. In the light of such a theory, we must also admit heredity as a cause.

Having regarded rickets as a disease of faulty nutrition, it will now be interesting to consider its pathological features. The most noticeable lesions are observed in the bones and teeth, but the muscles, nerves, skin, and in fact all the tissues of the body, may become involved. The old idea which prevailed in medicine ascribed the deformity of the spinal column, which commonly occurs in an early stage of rickets, and is a prominent feature, to softening of the vertebral bones. Hence the origin of the name rachitis from rachis, the spine. We now know, however, that this deformity proceeds from muscular weakness only. Nevertheless, there does occur in the course of the disease softening of the bones, from which, as a result of pressure applied in various directions, arise deformities. This softening of the bones is due to a deficient deposit of lime-salts in the process of ossification, and not to any subsequent absorption of these salts. The present view in regard to the pathology of the disease, as stated by Dr. Barlow, of London, who is perhaps one of the highest authorities on the subject, is that there is an inordinate production of immature osteoblasts or bone cells at the seat of growth of the bones. These crowd upon one another in such a rapid manner that there is not only incomplete ossification taking place, but in some parts of the growth premature ossification as well. This pathological feature leads to the belief that the young bone cells are over-stimulated in growth by some element in the blood acting as an irritant to the nutrition of the bone cell. One theory attributes this irritant subject to lactic acid. Exactly what it is we do not as yet know. Recent advances made in the study of the blood may possibly tend to throw much light on this point, and it may be found that hemogenesis is an all important factor in the etiology of rickets.

The overgrowth of imperfect bone cells is most strikingly shown near the ends of the long bones at their point of growth known as the epiphyses, occurring as swellings, which may be easily recognized. In the bones of the skull the openings, as a rule, close late, but may also close too early, and there are sometimes found soft spots in the occipital region along the line of the sutures.

Next to the lesions of the osseous system, the teeth in their development and subsequent condition present the most noticeable feature of rickets. The temporary teeth are late in development, and the intervals between the appearance of the various groups are longer and more irregular than the normal average. So characteristic is this feature that I have come to be suspicious of the existence of rickets in any infant who has reached the first year of life without showing a tooth. I am fully aware that in holding this view I am at variance with other good and competent observers, but so far as my memory serves me, a close investigation has always confirmed my suspicions. It may be interesting at this point to call attention to the observations made by Dr. A. Brothers of this city, in an analysis of over 500 cases in infants where the various stages of dentition were recorded, that in those infants affected with marasmus or acute malnutrition from digestive causes only, out of eight observed the first tooth appeared at an average of five months, the further progress of eruption, however, being somewhat delayed. I mention this as showing a further contrast of marasmus with rickets, even upon the development of the teeth. (The cross growth of teeth, or their irregular position in the alveolar cavity, has likewise been attributed to rickets.)

The most interesting feature, however, regarding the teeth in rickets is one that concerns the dentist more than the general practitioner of medicine. I refer to the tendency to early decay of the temporary teeth, and less frequently the permanent teeth. This decay I have often observed occurring in the central incisors even before the eruption of later groups. It was in several instances manifested as a distinct notch in the crown of one or both upper central incisors. In older children many teeth may be frequently found decaying rapidly at one time, as if they had crumbled away. The notched appearance of the upper central incisors occurring in the temporary teeth should not be confused with the same condition occurring in the permanent teeth. It is the latter that is known as the Hutchinson tooth, and is a stigma of inherited syphilis.—*Dental Cosmos.*

